# Analysis and prevention of microbial degradation of shadow puppetry artifacts preserved in the National Shadow Puppetry Museum in Chengdu

**DOI:** 10.3389/fmicb.2025.1611451

**Published:** 2025-06-03

**Authors:** Yu Wang, Yuanyuan Wang, Zhiqian Guan, Zeao Wang, Yangbo Duan, Chen Min, Yuhan Zhong, Lilong Hou, Jiao Pan

**Affiliations:** ^1^Key Laboratory of Archaeomaterials and Conservation, Ministry of Education, Institute of Cultural Heritage and History of Science and Technology, University of Science and Technology Beijing, Beijing, China; ^2^School of Archaeology and Museology, Sichuan University, Chengdu, China; ^3^Chengdu Museum (National Shadow Puppetry Museum in Chengdu), Chengdu, China; ^4^Department of Microbiology, College of Life Sciences, Nankai University, Tianjin, China

**Keywords:** shadow puppetry artifacts, microbial degradation, microbial community, antimicrobial agents, cultural relics preservation

## Abstract

Shadow puppetry, an integral and crucial component of China’s intangible cultural heritage, currently faces the significant threat of microbial degradation. This is primarily due to the organic materials used in its artifacts. This study centers on the shadow puppets housed in the National Shadow Puppetry Museum in Chengdu. By employing Scanning Electron Microscopy (SEM) and high-throughput sequencing techniques, it has revealed a diverse array of co-existing microorganisms on the surfaces of these puppets. These include species from genera such as *Aspergillus*, *Streptomyces*, *Nocardiopsis*, *Pseudomonas*, and *Saccharopolyspora*, among others. Eleven microbial species were successfully isolated, wherein four were identified as predominant: *Pseudomonas* sp. WH. S-B1, *Streptomyces* sp. WH. S-B2, *Nocardiopsis* sp. WH. S-B6, and *Aspergillus fumigatus* WH. S-F2. Notably, these four strains demonstrated the ability to degrade collagen. The antimicrobial experiment results indicated that 0.3% isothiazolinone-based antimicrobial agents BC01 and 50 mg/mL carvacrol exhibited a certain degree of antimicrobial activity against these predominant strains. Overall, this research provides a robust foundation for the conservation of shadow puppet artifacts. It does so by thoroughly analyzing the mechanisms of microbial degradation and screening effective antimicrobial agents.

## 1 Introduction

Shadow puppetry, a quintessential medium of China’s intangible cultural heritage, is a multifaceted art form that seamlessly integrates painting, carving, opera, and the esthetics of light and shadow (Liang et al., 2023; Huang et al., 2015; Yang et al., 2024). The artifacts are primarily crafted from animal hides, such as cowhide and sheepskin, through traditional processes encompassing tanning, carving, and dyeing (Duan et al., 2024). Abundant in collagen and organic dyes, these artifacts act as “living fossils,” meticulously documenting the evolution of ancient Chinese social culture, folk beliefs, and artisanal techniques (Lei et al., 2023; Li and Cao, 2021; Liu et al., 2019). The existing shadow puppetry artifacts from the Ming and Qing dynasties not only boast high artistic and esthetic value but also serve as irreplaceable physical evidence for the study of ancient social and cultural life (Prahmana and Istiandaru, 2021; Padovani, 2023; Coar and Usta, 2009). Nevertheless, due to the organic composition of shadow puppetry artifacts, they are highly vulnerable to damage caused by temperature and humidity fluctuations, pollutant erosion, and microbial activity during long-term preservation (Zhang et al., 2024). Among these threats, microbial-induced material degradation has become a critical concern endangering the preservation of these cultural relics. Conducting systematic research not only overcomes the empirical limitations of traditional cultural relic preservation but also provides theoretical frameworks for elucidating the degradation mechanisms of organic cultural heritage (Sun et al., 2024; Sterflinger and Piñar, 2013).

Recent investigations have demonstrated that microbial communities inhabiting the surface of shadow puppet artifacts can gradually deteriorate the collagen fiber network of leather. This occurs through the secretion of metabolic byproducts such as proteases and lipases, ultimately resulting in a loss of mechanical integrity and the detachment of pigment layers (Perini et al., 2019; Zugno et al., 2011; Strzelczyk et al., 1987). International research efforts have identified key degrading microbial groups. This includes fungi from the *Aspergillus* and *Penicillium* genera, as well as *Bacillus* bacteria. Their metabolic activities exhibit significant correlations with the pH and moisture levels of the leather (Sterflinger, 2010). The most recent research, utilizing metagenomic sequencing technology, has unveiled the spatial heterogeneity of microbial communities. It has revealed that microenvironments within the crevices of artifacts are particularly susceptible to the formation of synergistic degradation systems involving actinobacteria and fungi (Shi et al., 2020; Gadd et al., 2024; Wang et al., 2023).

Nevertheless, the current systematic research on the microbial deterioration of shadow puppet relics remains fraught with several limitations. Firstly, the dynamic fluctuations of microbial communities under varying environmental conditions have not been comprehensively explored. Secondly, existing preventive and control measures predominantly draw on the experiences from the conservation of other cultural relic types. As a result, their specificity and efficacy demand further in-depth investigation (Zhang et al., 2022). Conducting a holistic analysis of microbial deterioration in shadow puppet relics and formulating scientifically robust prevention and control strategies are of critical significance for the long-term conservation and cultural heritage succession of these invaluable artifacts.

This research is centered on the shadow puppets housed in the National Shadow Puppetry Museum in Chengdu. The majority of these shadow puppet cultural relics were donated by individuals from diverse backgrounds. Consequently, their preservation conditions vary significantly, some of these artifacts are afflicted with microbial contamination. Through painstaking investigation and sampling, a systematic examination was carried out on the microbial degradation processes occurring on the surfaces of these shadow puppet artifacts. Subsequently, appropriate antimicrobial agents were screened, thereby providing a foundation for the conservation and protection of shadow puppet artifacts.

## 2 Materials and methods

### 2.1 Sample collection and microbial investigation

**Figure 1:**
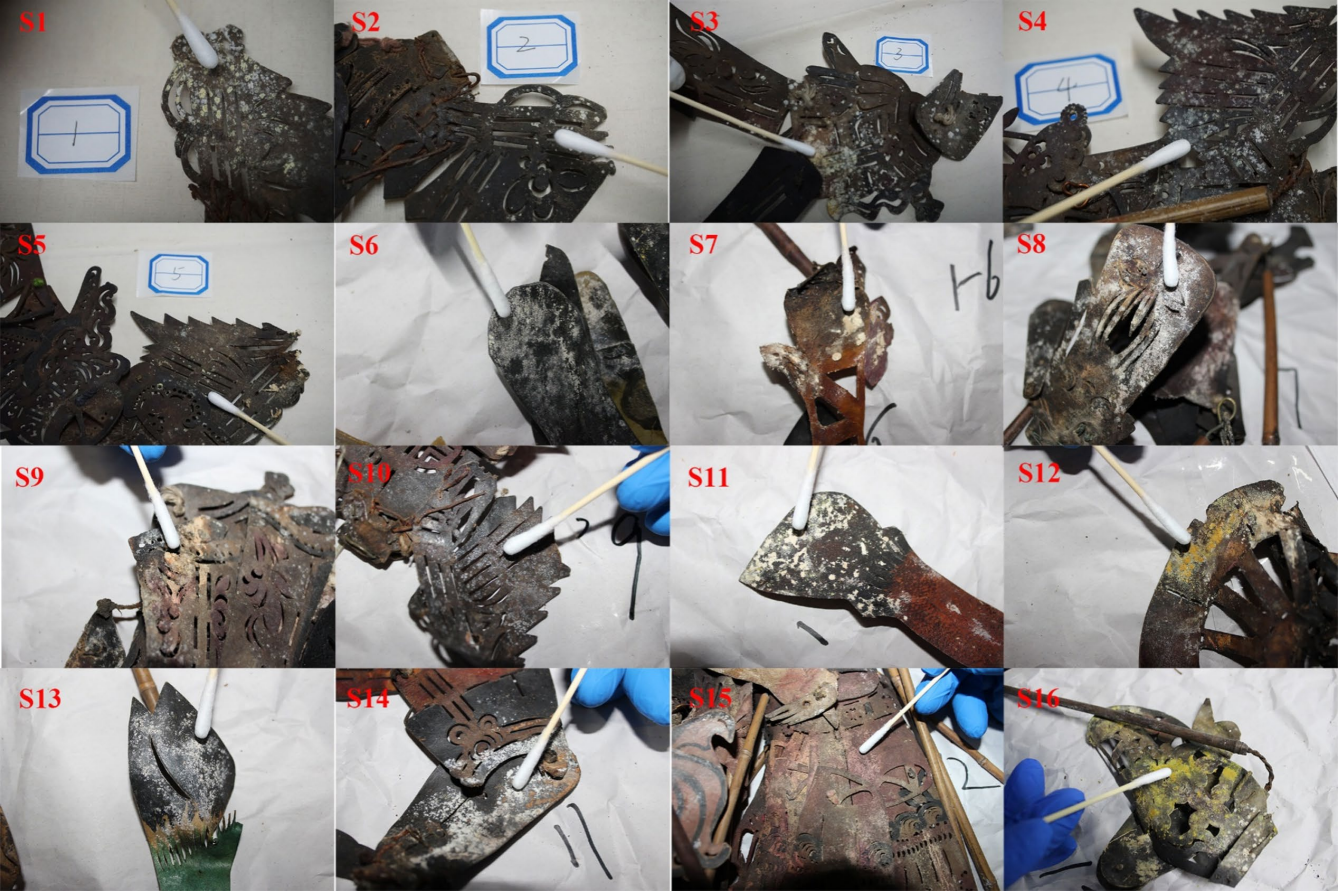
Microbial plaque on shadow puppet relics. S1–S5 were categorized as having mild contamination, S6–S10 exhibited moderate contamination, and S11–S16 showed severe contamination.

In October 2022 and March 2023, a microbiological survey was conducted on shadow puppet artifacts stored in the repository of the National Shadow Puppetry Museum in Chengdu. Crafted entirely from cowhide, these shadow puppet samples were sourced from various rural areas, primarily including Shaanxi, Sichuan, and Hebei. The majority of the shadow puppets date back to the period spanning from the Republic of China era to modern and contemporary times. The artifacts were housed in a climate-controlled warehouse. The temperature was maintained at 20 ± 2°C, with a daily variation of less than 2°C, while the humidity was kept at 50 ± 5%, with a daily fluctuation of less than 5%. The study detected microbial colonization on the surfaces of certain cultural relics (Figure 1). To evaluate the severity of microbial degradation, a grading system was established based on the colony coverage area. Samples S1–S5 were classified as having mild contamination, S6–S10 as moderate, and S11–S16 as severe. Microbial plaques were collected from the shadow puppet surface using sterile cotton swabs and then streaked onto Potato Dextrose Agar (PDA), Luria-Bertani (LB), and Gauze’s No. 1 media plates. These plates were transported to the laboratory for subsequent microbial isolation and purification. Meanwhile, surface plaques were carefully scraped with a sterile scalpel and placed into sterile EP tubes. These samples were transported on dry ice to the laboratory and stored at −80°C for subsequent amplicon sequencing and metagenomic analysis. During the survey, the ambient temperature in the repository was 18°C with a relative humidity of 56%.

### 2.2 Scanning electron microscope observation

Microbial samples were collected from the surfaces of shadow puppet artifacts and adhered to a substrate using carbon conductive adhesive. The samples were then dried in a desiccator. Once dried, each sample was mounted onto an SEM specimen stage. Gold sputtering was performed at a current of 24 mA for 300 s. Subsequently, the samples were examined using a SEM, and images were captured. The measurement parameters were set as follows: electron high tension (EHT) at 15.0 kV, working distance (WD) ranging from 9.5 to 10.7 mm, and magnification (Mag) between 2,000× and 10,000×.

### 2.3 Isolation and identification of culturable microorganisms

For the isolation and cultivation of fungi, PDA medium (1.2% potato extract, 2% glucose, 2% agar) was employed. LB medium (1% tryptone, 0.5% yeast extract, 1% NaCl, 2% agar, pH7.2) was utilized for bacterial isolation and cultivation. Gauze’s No.1 Medium (1% soluble starch, 0.2% KNO₃, 0.05% K₂HPO₄·3H₂O, 0.05% MgSO₄·7H₂O, 0.001% FeSO₄·7H₂O, 2% agar, pH7.5) served as the growth medium for actinomycetes. These microorganisms were incubated under specific conditions: fungi were cultured at 28°C for 5 days, bacteria at 37°C for 2 days, and actinomycetes at 28°C for 10 days. After the incubation period, all microbial colonies that developed on the media were systematically isolated and purified. Genomic DNA was then extracted from these pure cultures and amplified using polymerase chain reaction (PCR) to generate PCR products. For fungal amplification, the primers ITS1 (5′-TCCGTAGGTGAACCTGCGG-3′) and ITS4 (5′-CCTCCGCTTATTGATATGC-3′) were used, while the bacterial amplification employed primers 341F (5′-CCTACGGGAGGCAGCAG-3′) and 907R (5′-CCCCGTCAATTCATTTGAGTTT-3′). The PCR reaction conditions remained uniform across all samples, with the following reaction program: an initial denaturation step at 98°C for 3 min, followed by 33 cycles of 98°C for 10 s, 57°C for 10 s, and 72°C for 50 s. A final extension step was conducted at 72°C for 5 min, and the samples were held at 4°C. The resulting PCR products were sent to GENEWIZ (Beijing, China) for sequencing. Subsequently, the sequence homology of the amplified fragments was analyzed using tools available on the National Center for Biotechnology Information (NCBI) platform. The obtained sequences have been deposited in GenBank for public access and further research.

### 2.4 Amplicon sequencing and metagenomic sequencing analysis

Samples S1–S10 exhibited relatively mild microbial contamination, and their microbial compositions were analyzed using amplicon sequencing. Conversely, samples S11–S16 presented more severe microbial challenges. To comprehensively examine both their microbial communities and the leather degradation processes mediated by these microorganisms, metagenomic sequencing was employed. Total genomic DNA was extracted using the DNeasy PowerSoil Pro Kit (QIAGEN, Germany; Cat. No. 47014), strictly following the manufacturer’s protocols. The extracted DNA samples were then submitted to NovoMagic Technology Co., Ltd. (Beijing, China) for sequencing. The raw sequencing data are publicly available in the NCBI Sequence Read Archive (SRA) under the study accession number PRJNA1231699 and PRJNA1230850.

### 2.5 Detection of microbial collagen degradation ability

When microorganisms were inoculated into a medium where gelatin served as the sole nitrogen source (comprising 1% gelatin, 1% glucose, 0.1% KH_2_PO_4_, 0.05% MgSO_4_·7H_2_O, 0.05% NaCl, 2% agar), their growth on this medium indicated their capacity to degrade collagen (Osathanunkul et al., 2013; Fuente et al., 2024).

### 2.6 Antimicrobial experiment

Building on previous experimental findings and relevant literature (Wang et al., 2024; Li et al., 2025), the antimicrobial agents selected for this study were the isothiazolinone-based agent BC01 and carvacrol, a principal component of a specific plant essential oil (Table 1). The experimental procedure was as follows: First, microorganisms were uniformly spread across the appropriate culture medium. Subsequently, four sterile filter paper disks, each with a diameter of 7 mm, were placed on the medium’s surface. Then, 5 μL of each antimicrobial agent, along with the negative control solution, were separately dispensed onto the filter paper disks. Finally, the inoculated culture medium was transferred to the corresponding incubator for cultivation. The presence and size of inhibition zones were observed to assess the antimicrobial efficacy of the tested agents.

**Table 1:** Antimicrobial agents used in the study.

Antimicrobial agents	Main components	Concentration	Solvent	Manufacturer
BC01	3% Isothiazolinone	0.3% (m/v)	H_2_O	Beijing Baicheng Wangda Biotechnology Co., Ltd.
Carvacrol	Carvacrol	50 mg/mL	DMSO	Macklin Biochemical Technology Co., Ltd.

## 3 Results

### 3.1 SEM results of samples from the surface of shadow puppet cultural relics

**Figure 2:**
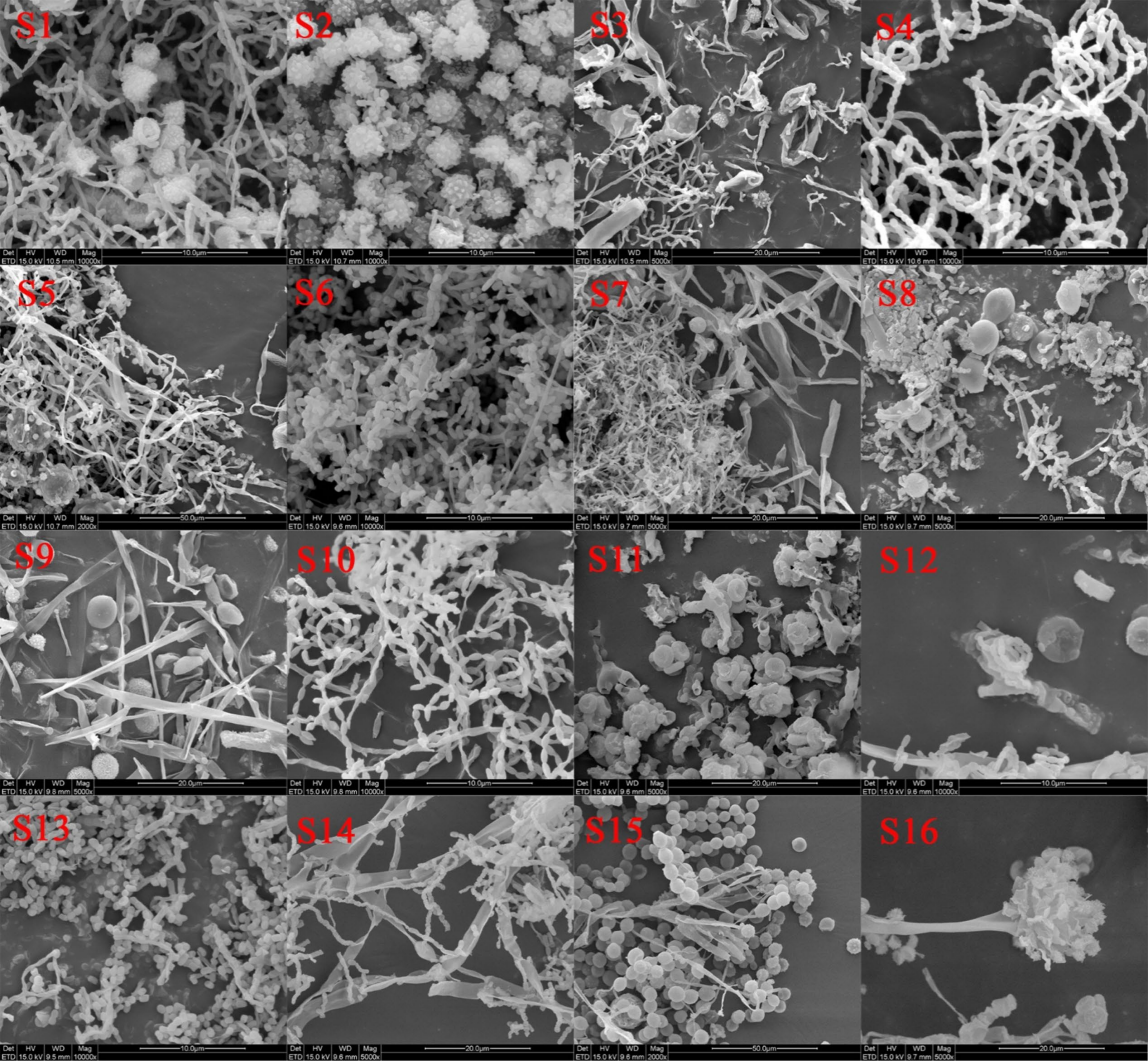
SEM results of samples from the surface of shadow puppet cultural relics.

SEM analysis provided conclusive evidence for the presence of microbial hyphae and spores (Figure 2). Significantly, both fungal and actinomycete species were simultaneously detected in multiple samples, including S1, S2, S7, and S14. This finding indicated that diverse microbial community inhabited the surface of shadow puppet cultural relics. These results strongly suggested that the visible microbial colonies on the artifacts are the result of complex synergistic interactions among multiple microbial species, rather than the growth of a single organism. In addition to microbial structures, SEM imaging also revealed the presence of insects on the surfaces of the shadow puppets (Supplementary Figure S1). These insects, when crawling on the puppets, can inadvertently transport microbial hyphae and spores, thereby facilitating the spread and exacerbating the contamination of these cultural artifacts by microorganisms. This biotic interaction represents a significant threat to the long—term preservation of shadow puppet cultural relics.

### 3.2 Microbial composition on the surface of the shadow puppet cultural relics

**Figure 3:**
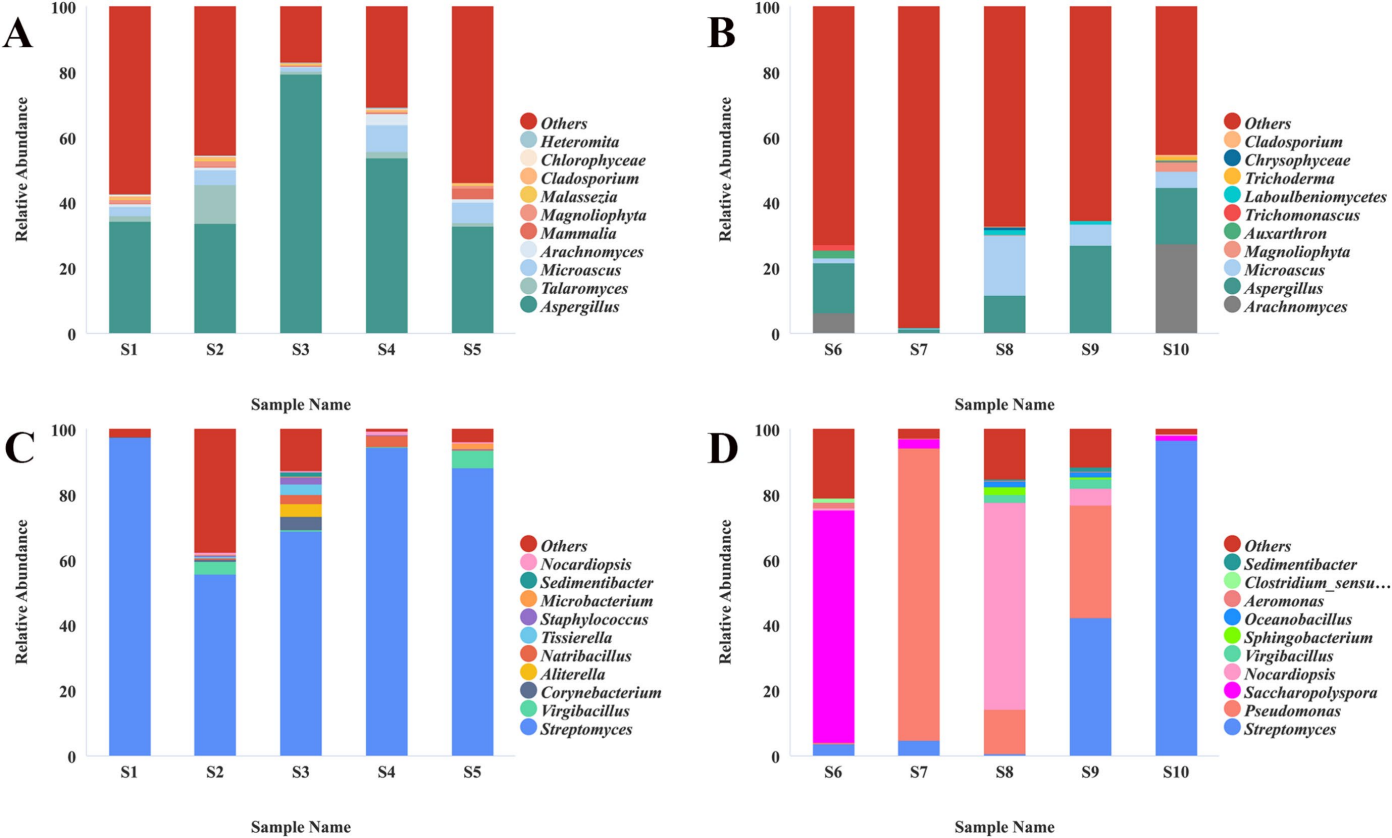
The relative abundance of microorganisms on the surface of shadow puppet cultural relics. **(A)** Eukaryotes, sample S1–S5; **(B)** eukaryotes, sample S6–S10; **(C)** prokaryotes, sample S1–S5; **(D)** prokaryotes, sample S6–S10.

**Figure 4:**
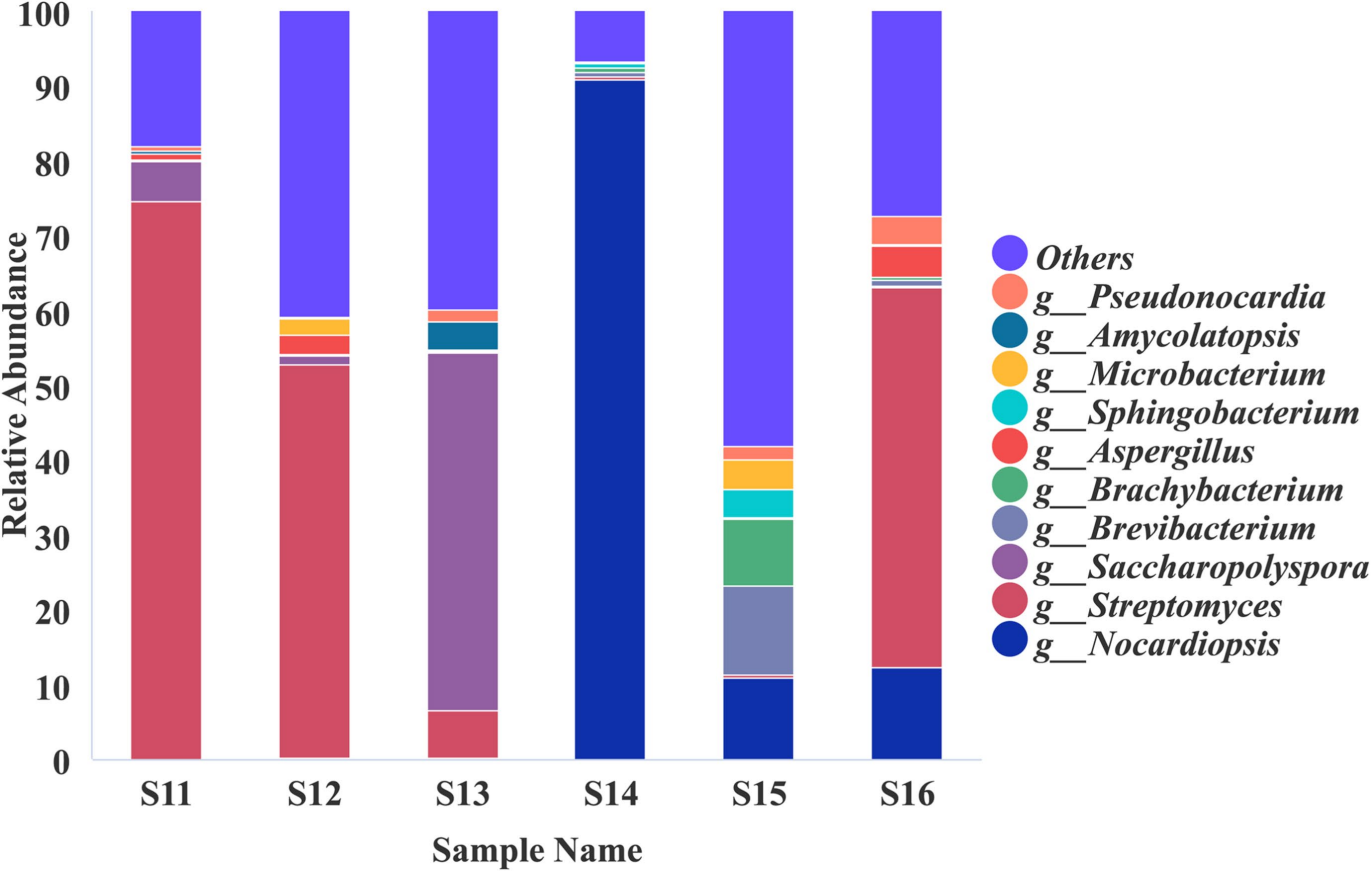
Relative abundance of microorganisms at the genus level on the surface of the Sample S11–S16.

High-throughput sequencing analysis of samples S1–S16 (Supplementary Figures S2–S4) revealed the presence of diverse microbial communities. Fungi predominantly belonging to the phylum Ascomycota and bacteria primarily classified under Actinobacteria were the dominant taxonomic groups on the surfaces of these cultural relics (Supplementary Figures S2–S4). Specifically, Firmicutes accounted for 14.22% of the prokaryotic community in samples S1–S5 (Supplementary Figure S2B), Proteobacteria constituted 30.68% of prokaryotes in samples S6–S10 (Supplementary Figure S3B), and Pseudomonadota represented 5.05% of the total microbial population in samples S11–S16 (Supplementary Figure S4). In-depth taxonomic profiling demonstrated significant variations in the dominant microbial communities across different shadow puppet artifacts. Samples S1–S5 shared similar microbial profiles, with *Aspergillus* (fungi) and *Streptomyces* (bacteria) as the predominant genera (Figures 3A,C). In contrast, samples S6–S10 exhibited notable heterogeneity: *Aspergillus* and *Pseudomonas* were enriched in S6; *Pseudomonas* was the dominant genus in S7; S8 was characterized by the co-occurrence of *Aspergillus*, *Microascus*, *Pseudomonas*, and *Nocardia*, S9 featured elevated abundances of *Aspergillus*, *Streptomyces*, and *Pseudomonas*; and S10 was dominated by *Aspergillus*, *Arachnomyces*, and *Streptomyces* (Figures 3B,D). Metagenomic sequencing of samples S11–S16 further identified prokaryote-dominated communities, with *Nocardiopsis*, *Streptomyces*, *Saccharopolyspora*, *Brevibacterium*, and *Brachybacterium* as the key taxonomic representatives (Figure 4). These findings highlight the complex and diverse microbial ecosystems associated with shadow puppet cultural relics, which may have implications for their conservation and preservation.

### 3.3 Database annotation

**Figure 5:**
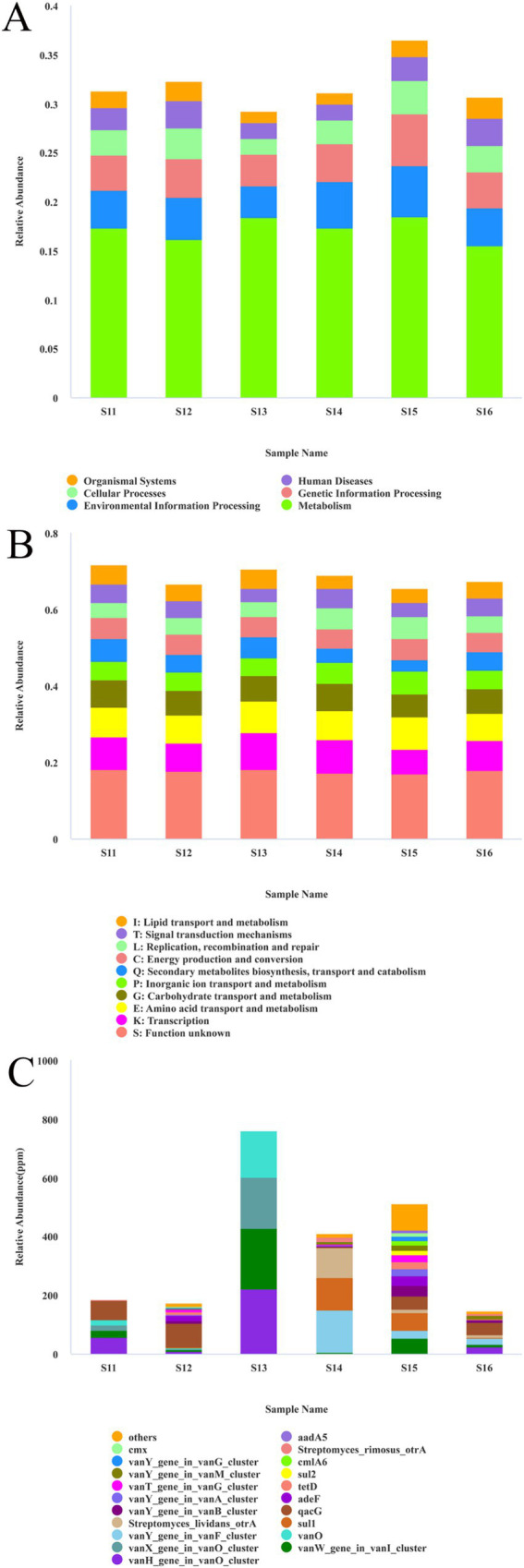
Database annotation results of each functional annotation: **(A)** relative abundance of KEGG database at level 1 in metagenome sequencing; **(B)** relative abundance of eggNOG database at level1 in metagenome sequencing; **(C)** relative abundance of CARD database in metagenome sequencing.

To elucidate the potential degradation mechanisms of microorganisms on shadow puppets, the sequencing results were annotated across multiple databases. Analysis using the Kyoto Encyclopedia of Genes and Genomes (KEGG) database revealed that metabolic pathways represented a significant proportion in each sample, ranking among the primary functional categories. This finding underscores the critical role of metabolic functions within these microbial communities (Figure 5A). Functional annotation based on the eggNOG database demonstrated that, while there was a degree of similarity in the overall composition of functional categories across samples, notable variations in specific proportions were observed. Transcription, amino acid transport, metabolism, and related categories were consistently highly represented in multiple samples. For example, in sample S11, the category of unknown function was the most abundant, followed by transcription, amino acid transport and metabolism. In contrast, sample S12 showed elevated proportions of carbohydrate transport and metabolism, inorganic ion transport and metabolism, among others (Figure 5B). Examination of the Comprehensive Antibiotic Resistance Database (CARD) revealed significant differences in the relative abundance and composition of antibiotic-resistance genes. Sample S13 exhibited the highest relative abundance, primarily attributable to genes such as *vanW* gene in *vanI* cluster (Drug Class: glycopeptide antibiotic), *vanX* gene in *vanO* cluster (Drug Class: glycopeptide antibiotic), and *vanY* gene in *vanA* cluster (Drug Class: glycopeptide antibiotic). Sample S15 also had a relatively high relative abundance with a complex gene composition, including genes such as *aadA5* (Drug Class: aminoglycoside antibiotic). In contrast, samples S11, S12, and S16 showed lower relative abundances, characterized by dispersed gene components and minor proportions. Sample S14 displayed a moderate relative abundance, predominantly composed of genes like the *vanY* gene in *vanG* cluster (Drug Class: glycopeptide antibiotic). These results illustrated the distinct distribution patterns of antibiotic-resistance genes across samples, providing essential data for understanding the dissemination and evolution of these genes in different environmental contexts (Figure 5C).

### 3.4 Isolation and identification of dominant microorganisms

**Figure 6:**
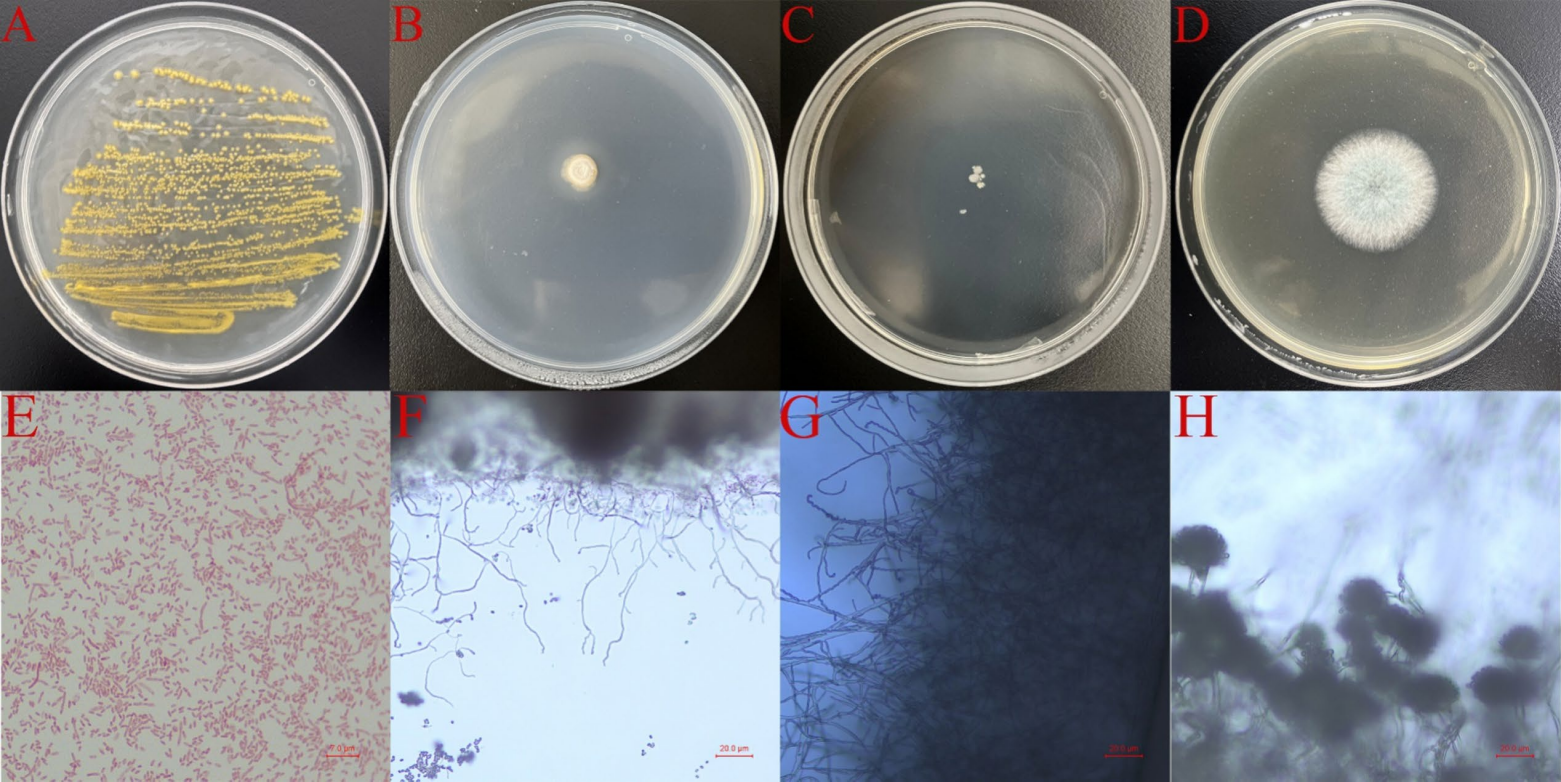
The morphology of four microorganisms on the culture medium and under the microscope. **(A)** *Pseudomonas* sp., WH. S-B1, LB medium; **(B)** *Streptomyces* sp., WH. S-B2, Gauze’s No.1 medium; **(C)** *Nocardiopsis* sp., WH. S-B6, Gauze’s No.1 medium; **(D)** *Aspergillus fumigatus*, WH. S-F2, PDA medium; **(E)** WH. S-B1; **(F)** WH. S-B2; **(G)** WH. S-B6; **(H)** WH. S-F2.

**Figure 7:**
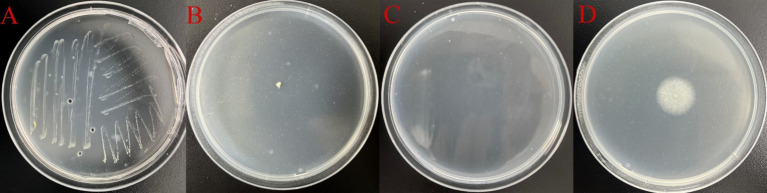
The four microorganisms on the gelatin medium. **(A)** *Pseudomonas* sp., WH. S-B1; **(B)** *Streptomyces* sp., WH. S-B2; **(C)** *Nocardiopsis* sp., WH. S-B6; **(D)** *Aspergillus fumigatus*, WH. S-F2.

Eleven distinct microbial species were successfully isolated and purified using standard microbiological culture techniques, including four bacterial strains, two actinomycete strains, and five fungal isolates (Table 2). Among these, four strains—*Pseudomonas* sp. (WH. S-B1), *Streptomyces* sp. (WH. S-B2), *Nocardiopsis* sp. (WH. S-B6), and *Aspergillus fumigatus* (WH. S-F2)—corroborated the findings from high-throughput sequencing data, confirming their status as predominant microorganisms on the shadow puppet artifacts (Figure 6). These four strains were thus selected for further in-depth investigation. Growth assays on media supplemented with gelatin as the sole nitrogen source demonstrated that *Pseudomonas* sp., *Streptomyces* sp., *Nocardiopsis* sp., and *Aspergillus fumigatus* were capable of utilizing gelatin for growth (Figure 7). This phenotypic characteristic strongly suggests that these four strains possess the metabolic potential to degrade collagen, a major structural protein component of shadow puppets. These results provide crucial insights into the biochemical mechanisms underlying the deterioration of shadow puppet cultural relics.

**Table 2:** Molecular identification of strains isolated from the shadow puppet artifacts.

Microorganisms	Genus	Genbank number
WH. S-B1	*Pseudomonas* sp.	PV569711
WH. S-B2	*Streptomyces* sp.	PV569712
WH. S-B3	*Lysinibacillus fusiformis*	PV569713
WH. S-B4	*Brachybacterium* sp.	PV569714
WH. S-B5	*Bacillus subtilis*	PV569715
WH. S-B6	*Nocardiopsis* sp.	PV569716
WH. S-F1	*Penicillium aethiopicum*	PV576011
WH. S-F2	*Aspergillus fumigatus*	PV576012
WH. S-F3	*Cladosporium cladosporioides*	PV576013
WH. S-F4	*Penicillium oxalicum*	PV576014
WH. S-F5	*Gymnoascus* sp.	PV576015

### 3.5 Sensitivity of dominant microorganisms to different antimicrobial agents

**Figure 8:**
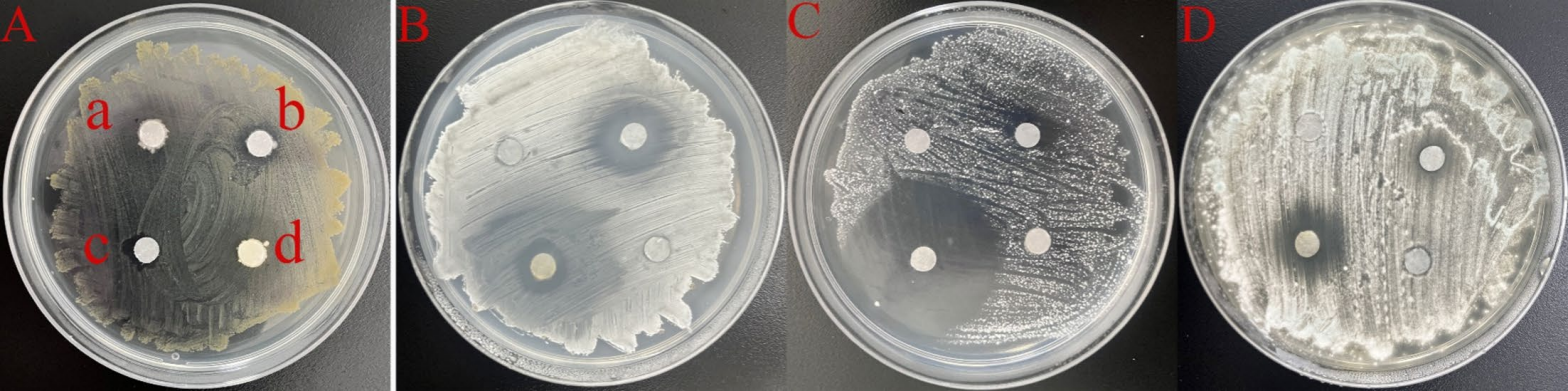
The antimicrobial effects of 0.3% BC01 and 50 mg/mL carvacrol. **(A)** DMSO; **(B)** 50 mg/mL carvacrol; **(C)** 0.3% BC01; **(D)** H_2_O. Among them, DMSO and H_2_O served as the negative controls. **(A)** *Pseudomonas* sp., WH. S-B1; **(B)** *Streptomyces* sp., WH. S-B2; **(C)** *Nocardiopsis* sp., WH. S-B6; **(D)** *Aspergillus fumigatus*, WH. S-F2.

To identify effective antimicrobial agents with minimal adverse effects on cultural relics, two candidates were selected for laboratory testing: BC01, an isothiazolinone-based antimicrobial agent currently utilized in cultural heritage conservation, and carvacrol, a main component of certain plant essential oils. The antimicrobial assay results indicated that 0.3% BC01 and 50 mg/mL carvacrol exhibited limited inhibitory activity against *Pseudomonas* sp. (WH. S-B1). Conversely, BC01 demonstrated notable antibacterial and antifungal effects against *Streptomyces* sp. (WH. S-B2), *Nocardiopsis* sp. (WH. S-B6), and *Aspergillus fumigatus* (WH. S-F2), with the most pronounced effect observed against *Nocardiopsis* sp. Carvacrol showed significant inhibitory efficacy against *Streptomyces* sp. and *Nocardiopsis* sp. (Figure 8). These findings suggest that BC01 and carvacrol hold potential for application in the conservation of shadow puppet cultural relics, warranting further investigation into their long-term efficacy and compatibility with artifact materials.

## 4 Discussion

This study represents the first investigation into the microbial degradation of shadow puppet cultural relics. The SEM analysis provided conclusive evidence for the co-existence of diverse microbial species, including fungi and actinomycetes, on the surface of these artifacts. This finding aligns with previous research (Zhang et al., 2022), which indicates that the degradation of cultural heritage is often the result of synergistic interactions among multiple microorganisms. High-throughput sequencing further elucidated the complex microbial communities associated with the shadow puppets. The prevalence of Ascomycota among the fungal taxa and Actinobacteria within the bacterial groups across all samples (S1–S16) highlights their potential significance in the degradation process. Distinct patterns of microbial dominance were observed: Firmicutes were relatively abundant in S1–S5, Proteobacteria dominated in S6–S10, and Pseudomonadota were prevalent in S11–S16. These variations likely reflect differences in the microenvironmental conditions of individual artifacts, such as variations in preservation history, exposure to pollutants, and the chemical composition of the leather resulting from diverse tanning and dyeing processes. The identification of dominant genera, including *Aspergillus*, *Streptomyces*, *Pseudomonas*, and *Nocardiopsis,* is consistent with international research on the microbial degradation of organic materials (Creangă, 2010; Piñar et al., 2020; Bicchieri et al., 2019; Pangallo et al., 2010). The ability of four selected strains (*Pseudomonas* sp., *Streptomyces* sp., *Nocardiopsis* sp., and *Aspergillus fumigatus*) to grow on media containing gelatin as the sole nitrogen source strongly suggests their potential to degrade collagen, a major structural component of leather artifacts. Collagen degradation compromises the mechanical strength and structural integrity of shadow puppets, a critical factor in their deterioration. Previous studies have shown that microorganisms secrete proteases and lipases, which can break down collagen fiber network and pigment layers (Zugno et al., 2011; Israelroming et al., 2012). In summary, the identification of these degradation- associated microorganisms and their functional capabilities provides fundamental insights into the mechanisms underlying the deterioration of shadow puppet cultural relics.

Previous international studies have consistently identified *Aspergillus* and *Penicillium* as dominant fungal genera and *Bacillus* as a key bacterial genus involved in the degradation of various organic cultural relics (Yu et al., 2022; Du et al., 2024; Zhang et al., 2021; Lao et al., 2024; Shao et al., 2023; Zhao et al., 2023; Zhang et al., 2023; Huang et al., 2021). In contrast, while *Aspergillus* was also frequently detected in this study, other genera including *Pseudomonas*, *Streptomyces*, and *Nocardiopsis* emerged as significant contributors to the degradation of shadow puppet relics. This divergence in microbial composition can be attributed to the unique material properties of shadow puppet, which are predominantly crafted from animal hides subjected to specialized tanning and dyeing processes. These manufacturing techniques create a distinct ecological niche that selects for specific microbial taxa, differentiating the microbial communities associated with shadow puppets from those found on other organic artifacts such as wooden objects or paper documents. Recent advancements in metagenomic sequencing have highlighted the spatial heterogeneity of microbial communities with cultural relics (Gadd et al., 2024; Wang et al., 2023). Our study supports this emerging paradigm, as significant variations in microbial composition were observed across different shadow puppet samples (S1–S16). Although the spatial heterogeneity of microenvironments within shadow puppets, particularly in creases and folds where actinobacteria and fungi often form synergistic degradation systems, was not explicitly investigated in this study, it represents a promising avenue for future research. By elucidating these similarities and differences with previous research, this study contributes to a more comprehensive understanding of microbial—mediated degradation processes in organic cultural heritage, thereby facilitating the development of targeted conservation strategies. In this study, the antibacterial agents 0.3% BC01 and 50 mg/mL carvacrol demonstrated promising *in-vitro* bacteriostatic activity against the four selected microbial strains. However, their long-term efficacy and safety in actual cultural heritage conservation scenarios remain uncertain. Several critical aspects require further investigation. Firstly, potential chemical interactions between these agents and the leather materials of shadow puppets may occur. Such interactions could lead to material degradation, color change, or other physical alterations over time, potentially compromising the structural integrity of the artifacts. Secondly, the impact of these agents on the esthetic and historical value of shadow puppets needs to be carefully evaluated. Any treatment should preserve the unique characteristics and historical significance of these cultural relics without causing unintended damage. Furthermore, the optimal application methods for ensuring uniform distribution and sustained protection of these antibacterial agents have not been explored in this study. Previous research (Chen et al., 2024; Xu et al., 2020) has emphasized that the application techniques play a crucial role in the effectiveness of conservation treatments. Inadequate application could result in uneven protection, leaving certain areas of the artifacts vulnerable to microbial attack. In conclusion, while the initial findings on the antibacterial properties of BC01 and carvacrol are encouraging, comprehensive studies on their long-term performance, material compatibility, and appropriate application methods are essential before they can be safely and effectively implemented in the conservation of shadow puppet cultural relics.

## 5 Conclusion

This study investigated shadow puppets in the National Shadow Puppetry Museum in Chengdu, identifying diverse co-existing microorganisms on their surfaces and isolating four predominant collagen-degrading strains. Antimicrobial experiments showed that 0.3% isothiazolinone-based BC01 and 50 mg/mL carvacrol had antimicrobial effects to a certain extent. This research provides a basis for shadow puppet artifact preservation against microbial degradation.

## Data Availability

The datasets presented in this study can be found in online repositories. The names of the repository/repositories and accession number(s) can be found in the article/Supplementary material.
