# Supplementary material for: Analysis and prevention of microbial degradation of shadow puppetry artifacts preserved in the National Shadow Puppetry Museum in Chengdu
Source: Front Microbiol. 2025 Jun 3;16:1611451. doi: 10.3389/fmicb.2025.1611451 (PMC12170658; doi:10.3389/fmicb.2025.1611451)
Supplement: Supplementary file 1 [file Table_1.docx]

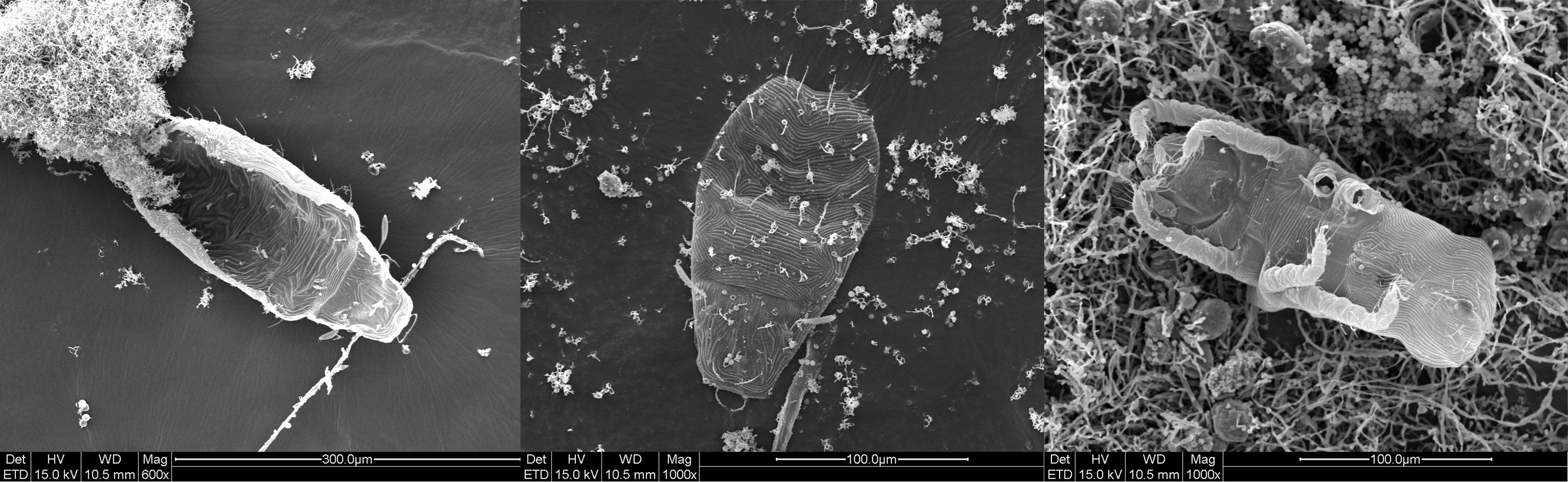


**Figure S1.** SEM results.


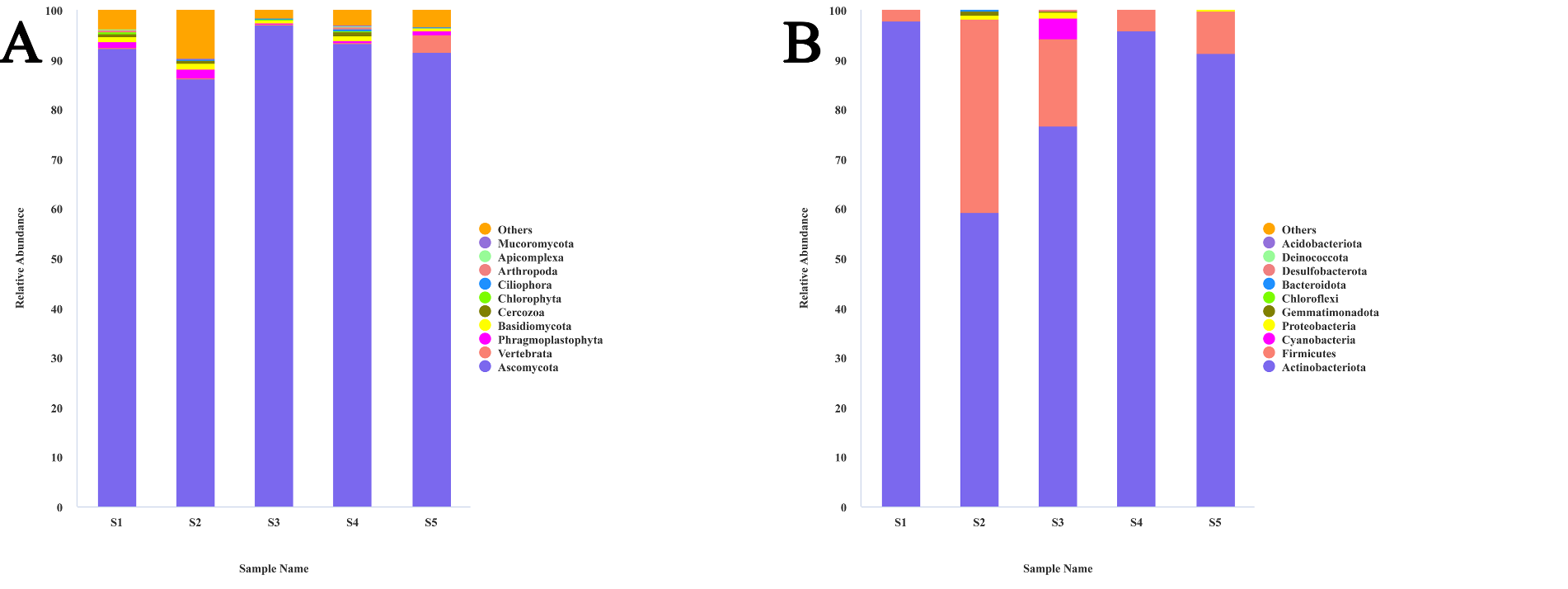


**Figure S2.** Relative abundance of eukaryotes (A) and prokaryotes (B) at the phylum level on the surface of the Sample S1-S5.


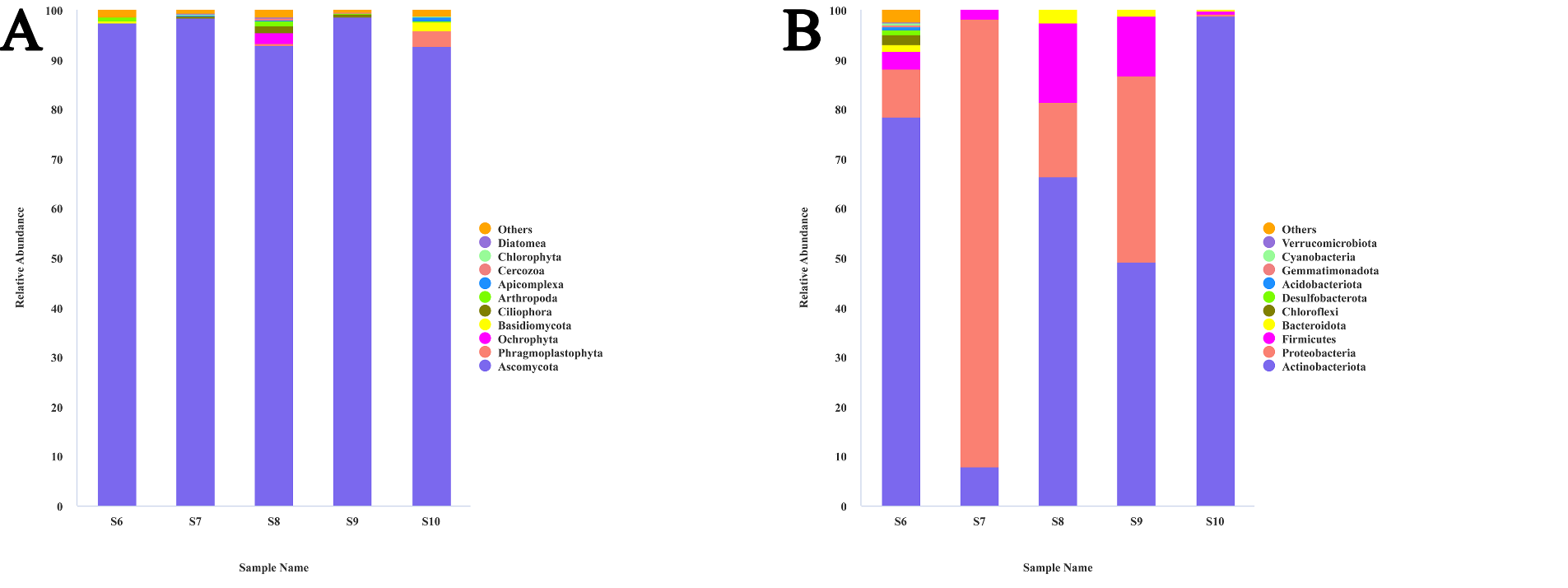


**Figure S3.** Relative abundance of eukaryotes (A) and prokaryotes (B) at the phylum level on the surface of the Sample S6-S10.


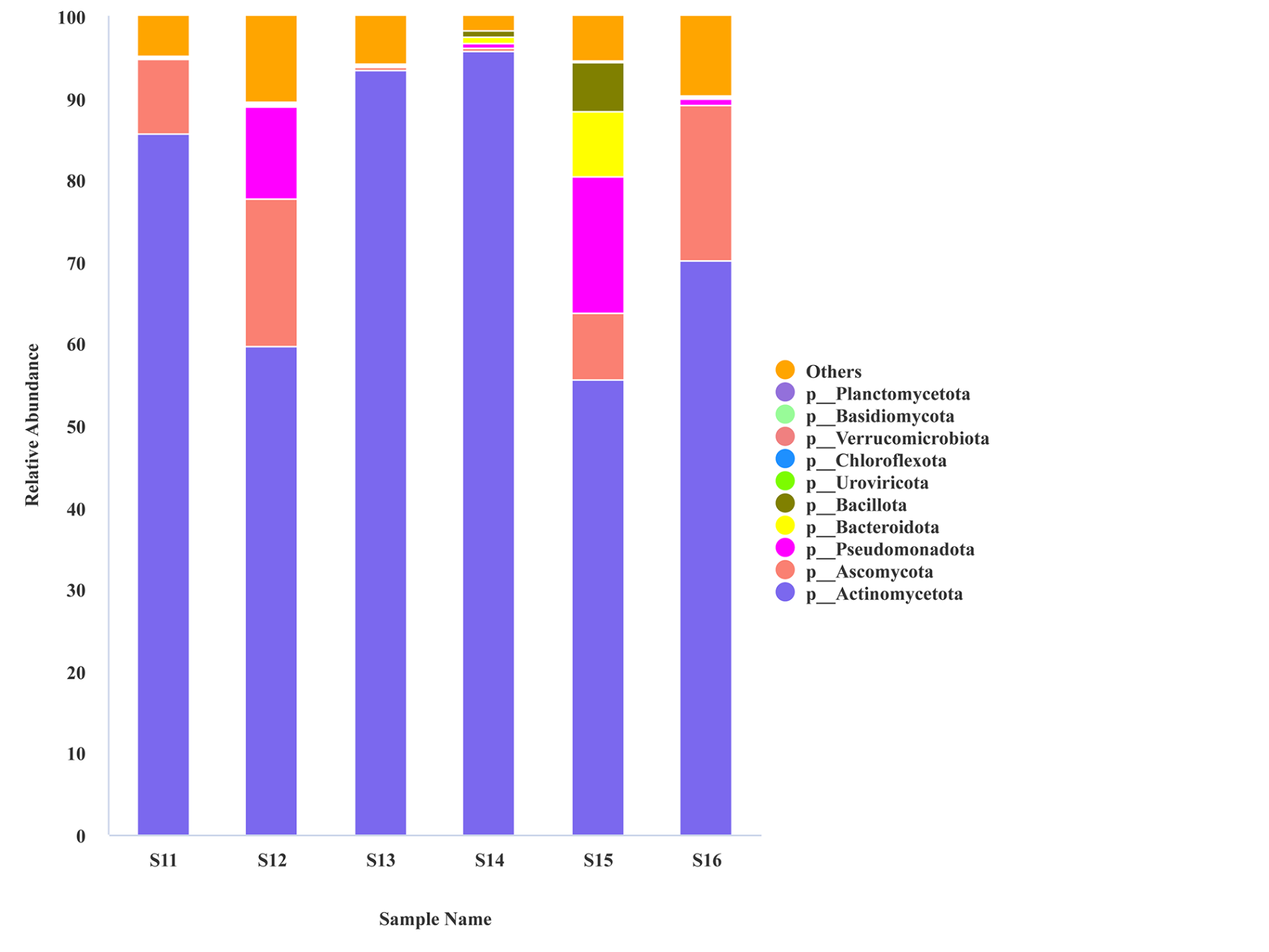


**Figure S4.** Relative abundance of microorganisms at the phylum level on the surface of the Sample S11-S16.
